# Sociometric Popularity, Perceived Peer Support, and Self-Concept in Adolescence

**DOI:** 10.3389/fpsyg.2020.594007

**Published:** 2020-11-26

**Authors:** Arantza Fernández-Zabala, Estibaliz Ramos-Díaz, Arantzazu Rodríguez-Fernández, Juan L. Núñez

**Affiliations:** ^1^Developmental and Educational Psychology Department, Education and Sport Faculty, University of the Basque Country (UPV/EHU), Vitoria-Gasteiz, Spain; ^2^Department of Psychology, Sociology and Social Work, University of Las Palmas de Gran Canaria, Las Palmas de Gran Canaria, Spain

**Keywords:** social preference, perceived social support, general self-concept, sociometer theory, secondary education

## Abstract

The objective of this study is to analyze the role that peer support plays in the incidence relationships between sociometric popularity and general self-concept based on sociometer theory. A total of 676 randomly selected secondary school students from the Basque Country (49.6% boys and 50.4% girls) between 12 and 18 years of age (*M* = 14.32, DT = 1.36) participated voluntarily. All of them completed a sociometric questionnaire (SOCIOMET), the Family and Friends Support Questionnaire (AFA-R), and the Dimensional Self-concept Questionnaire (AUDIM-33). Several models of structural equations were tested. The results indicate that sociometric popularity is linked to self-concept through the perceived social support of peers. These results are discussed within the framework of positive psychology and its practical implications in the school context.

## Introduction

A high-quality education system should promote interpersonal skills as part of the official curricula with the aim both of contributing to optimum socio-emotional functioning and avoiding a negative impact on students’ well-being. Indeed, research has shown that students’ development of emotional problems are linked to negative experiences with peers, such as peer victimization, rejection, and neglect and a negative self-concept (Wang et al., 2016; Norrington, 2020). Problems in peer relationships can have a severe negative effect on individuals’ emotional health and self-concept as indicator of well-being (Norrington, 2020; Schwartz-Mette et al., 2020). At the same time, previous research indicates that peer support positively impacts children’s school experience and could function as school bullying prevention (Tzani-Pepelasi et al., 2019). Moreover, students consider crucial peer relationships to their wellbeing experiences in school (Blaskova and McLellan, 2018). It is clear that interpersonal relationships entail health benefits and prevents possible damages in adolescence.

### Popularity and Self-Concept

The study of popularity forms part of a field of psychological research focusing on the interpersonal relationships established during childhood and adolescence. Popularity could be defined as the order in which children and adolescents are classified in their respective peer groups, in accordance with a hierarchical criterion (Bukowski, 2011). The concept comprises two dimensions that are related to each other but at the same time are different (Ferguson and Ryan, 2019; van den Berg et al., 2020): sociometric popularity, or social preference (Andreou, 2006), which refers to the feeling of being loved and accepted by one’s peers; and perceived popularity, which refers to prestige, visibility and dominance within the peer group (Cillessen and Marks, 2011; Cillessen and Van den Berg, 2012). The term social acceptance has often been used as a synonym of popularity, even though in reality it is simply a dimension of social self-concept (Fernández-Zabala et al., 2016b), understood as the self-evaluation of the degree to which a person feels accepted and loved by the significant others in their life. Thus, popularity and social acceptance are not the same thing and should not be used as synonyms due to the terminological confusion that this may generate, given that the two terms refer to separate yet interrelated concepts.

Deriving from this association between popularity and social acceptance (understood as a dimension of social self-concept), diverse studies have found a significant relationship between popularity and self-esteem, although two opposing theories exist regarding the causal directionality of said relationship. The first is Sociometer Theory (SMT) (Leary and Baumeister, 2000), which is based on the precepts of symbolic interactionism, since it emphasizes how social and other interactions together make up self-concept. This means that a person’s self-concept emerges from interactions with others and reflects the characteristics attributed to that individual by others, along with their expectations and assessments (Cooley, 1902; Mead, 1934). In specific terms, SMT posits that self-esteem is like a sociometer, i.e., influenced by social feedback from others. Thus, self-esteem suffers and decreases during experiences in which the individual feels socially excluded and increases when they feel included in a social situation.

The second theory is the Self-Broadcasting Perspective (SBP) (Taylor et al., 2003; Swann et al., 2007; Zeigler-Hill et al., 2013), which argues the opposite effect, i.e., that high self-esteem may predict social inclusion, since self-esteem may guide people to interpret social signals in a more favorable manner. Thus, according to the SBP, positive self-evaluation should lead to an increase in popularity.

While previous research has found evidence to support both theories, most recent findings suggest that the sociometer theory is empirically superior (Neziek, 2001; Murray et al., 2003; Srivastava and Beer, 2005; Birkeland et al., 2014; Reitz et al., 2016), thus confirming the idea that a person’s popularity determines their level of self-esteem. Nevertheless, a deeper analysis of the results of these studies reveals two limitations linked to the way in which popularity is measured. Firstly, the fact that the studies used different measurement instruments makes it difficult to compare results; and secondly, the use of self-reported questionnaires to measure popularity may call the reliability of the results into question (Brown and Larson, 2009), since it has been shown that those who tend to see themselves in a very positive light have a particularly strong self-report bias (Sedikides and Gregg, 2008). It is therefore clear that the directionality of the relationship between popularity and self-concept requires further confirmation using much more objective instruments, such as those which measure sociometric popularity and which could be completed by both the individual and their peers.

### Perceived Peer Support and Self-Concept

Social feedback from peers, parents and teachers may be especially influential during adolescence, when the urge to configure one’s individual identity implies the challenge of integrating different information about the self in a global self-concept (Erikson, 1968). Moreover, while family relationships continue to have a strong influence (Fernández-Zabala et al., 2016a; Axpe et al., 2019; Bully et al., 2019), the peer group is the primary socializing context during adolescence, since friendships contribute to both emotional and social development during this crucial stage (Bagwell and Bukowski, 2018; Bukowski et al., 2020; Schwartz-Mette et al., 2020).

Perceived social support, understood as the subjective perception of the support and regard shown toward oneself by significant others (Lakey and Scoboria, 2005), is a variable which has been found to influence school adaptation, risk prevention during adolescence (Mishna et al., 2016; Ramos-Díaz et al., 2016), and self-concept (Kong et al., 2015; Magro et al., 2019). At a theoretical level, some authors have emphasized the importance of social support from peers as a key element of self-esteem (Mruk, 2006), since the positive interrelationship between peer support and self-perceptions is currently an unquestionable empirical fact (Marshall et al., 2014; Sarkova et al., 2014). Indeed, previous research suggests that social support has a significant influence on different personal and school adjustment indicators, through self-esteem and self-concept (Kong et al., 2012, 2015; Kong and You, 2013; Ramos-Díaz et al., 2016), and some authors even claim that perceived support from peers may help explain the significant associations observed between sociometric status and school adjustment (Wentzel, 2003; Bellmore, 2011). Together, all these studies constitute a sound theoretical basis for the hypothesis that peer support, sociometric status and self-concept are related to each other in some way, with self-concept perhaps being particularly susceptible to social comments from peers during adolescence, due to the aforementioned importance of peer relations during this developmental period (Brown and Larson, 2009).

### Popularity, Perceived Peer Support, and Self-Concept

Although several bivariate analyses have been carried out, no studies to date have analyzed the interrelationships which exist between popularity, perceived peer support and self-concept in a combined manner. Regarding the relationship between popularity and peer support during adolescence, the few studies which exist refer mainly to friendship (Nangle et al., 2003; Rose et al., 2004; Stotsky and Bowker, 2018), analyzing number of friends, reciprocal friendship and conflict, etc., rather than the support provided by said friends. The results indicate that popularity is positively associated with number of friends, with those adolescents classified as being more popular claiming to have more friends (Bukowski et al., 1996; Nangle et al., 2003; Rose et al., 2004; Stotsky and Bowker, 2018). However, although studies which have explored the association between popularity and peer relations do not refer specifically to the social support provided, it has been observed that students obtaining few positive nominations and many negative nominations by their peers perceive less support from them (Wentzel, 2003), and that perceived popularity has been linked to greater support from friends (Litwack et al., 2012).

Regarding the association between popularity and self-concept, recent findings indicate that perceived popularity may have a direct effect on global self-concept, with no mediation by friendships (Litwack et al., 2012). However, the role played by perceived support from peers in the association between popularity and self-concept has yet to be determined.

### The Present Study

**FIGURE 1:**
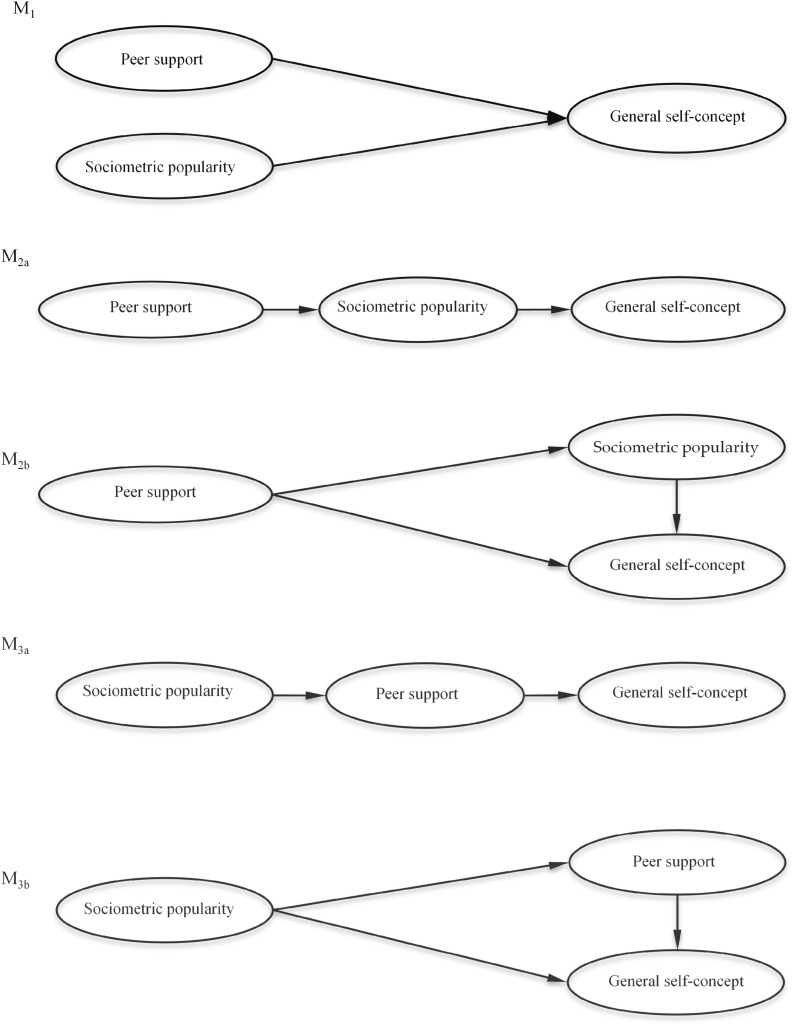
Hypothesized theoretical models.

Thus, the aim of this study is to analyze the role played by peer support in the relationship between sociometric popularity and general self-concept, taking Sociometer Theory (SMT), which posits that popularity influences self-concept (rather than the other way round) as our theoretical basis. The study therefore tests different structural models (see Figure 1) in order to determine whether peer support is a precursor or mediator variable for self-concept and if so, whether said mediation is full or partial. Firstly, it is proposed that peer support is a precursor variable for self-concept at the same level as sociometric popularity with no causal relationship between them (M_1_). Next, it is established that the peer support is a precursor variable of the self-concept, being mediated by the sociometric popularity in a full way (M_2a_) or partially (M_2b_). And finally, it is proposed that peer support mediates the relationship between sociometric popularity and self-concept in a full way (M_3a_) or partially (M_3b_).

## Materials and Methods

### Participants

Participants were 676 secondary students from different schools in the Autonomous Community of the Basque Country (ACBC) in Spain; 335 (49.6%) were boys and 341 (50.4%) were girls and all were aged between 12 and 18 years (*M*_age_ = 14.32; *SD* = 1.36). Schools were selected in accordance with a random process, with the final sample comprising 36 classes from 9 schools (five semi-private and four public). The families attending the schools had a mid-level socioeconomic and cultural status.

As shown in Table 1, participants were distributed throughout the different school years as follows: cycle 1 (years 1 and 2 of secondary school): 329 (48.7%); cycle 2 (years 3 and 4 of secondary school): 301 (44.5%); and cycle 3 (years 1 and 2 of the Spanish Baccalaureate): 46 (6.8%). Pearson’s chi-squared test revealed no differences in the distribution of each sex between the educational cycles [χ^2^(1) = 3.78, *p* = 0.151].

**TABLE 1:** Distribution of the sample by educational cycle and sex.

Sex	Educational cycle			Total
	Cycle 1	Cycle 2	Cycle 3	
Boys	170 (25.1%)	138 (20.4%)	27 (4.0%)	335 (49.6%)
Girls	159 (23.5%)	163 (24.1%)	19 (2.8%)	341 (50.4%)
Total	329 (48.7%)	301 (44.5%)	46 (6.8%)	676 (100%)

*χ^2^_(__1__)_ = 3.78, p > 0.05.*

### Measures

#### Sociometric Popularity

To calculate popularity it is used a sociometric questionnaire in which participants were asked to name three classmates they would choose as best friends and three classmates they would least like to have as friends. The responses were processed using the Sociomet computer program (González and García-Bacete, 2010) which analyzes class group relationships on the basis of the positive and negative nominations made by all students. For this study, the value calculated was the social preference index, which is the sum of the positive references to a subject, minus the sum of the negative references.

#### Perceived Peer Support

The Family and Friends Support Questionnaire (AFA-R; (González and Landero, 2014) was used to measure perceived peer group support. This questionnaire comprises 15 items which respondents rate on a 5-point Likert-type scale (1 = *never* to 5 = *always*). In this study, only the *support from friends* dimension (7 items, e.g., “Do you have a friend who shows you affection?”) was used. This dimension assesses the perceived availability of friends for talking and providing help, affection and support, as well as satisfaction with the support received. The internal consistency coefficient found in the original validation was = 0.89, and in this study it was = 0.85. Moreover, in the present study, the composite reliability coefficient (CFC) was 0.86, the average variance extracted (AVE) was.50, and the fit was acceptable: SBχ^2^(13) = 24.7119, *p* = 0.025 CFI = 0.989; TLI = 0.979; RMSEA = 0.037; RMSEA confidence interval 90% = 0.013–0.058.

#### General Self-Concept

This construct was evaluated using the Dimensional Self-Concept Questionnaire (AUDIM-33; (Fernández-Zabala et al., 2015). Although the full questionnaire comprises 12 scales (verbal academic self-concept, mathematical academic self-concept, physical ability, physical fitness, physical attractiveness, physical strength, honesty, emotional adjustment, autonomy, self-realization, social responsibility, and social competence), plus another for measuring general self-concept, only the general scale was used in this study. This scale comprises 5 items (e.g., “I feel like a lucky person”) which respondents rate on a 5-point Likert-type scale (1 = *false* to 5 = *true*). The reliability index for the general self-concept scale was 0.71 in this study, the CFC was 0.82 and the AVE was 0.50. Likewise, it also has a good fit: SBχ^2^(4) = 12.1052, *p* = 0.017; CFI = 0.987; TLI = 0.966; RMSEA = 0.055; RMSEA confidence interval 90% = 0.021–0.091.

### Procedure

After having received a favorable report from the University of the Basque Country’s Ethics Commission (CEISH/UPV-EHU, BOPV 32, EHAA, memory number M10/2015/076), stating that the study complied with the ethical values established for research with humans (informed consent, right to information, personal data protection, confidentiality guarantees, non-discrimination, non-remuneration and the right to withdraw at any time), nine schools, both public and semi-private (i.e., private but with some state funding) were selected using a simple random procedure from the official list of schools published by the Basque Government Department of Education. Two schools declined the invitation to collaborate in the project, and two new schools were selected to replace them, using the same method. Contact was made with the administrative team of each school to request their voluntary participation and to explain the nature of the research being carried out. After obtaining institutional permission and the informed consent of the families, the battery of questionnaires was administered collectively under the supervision of members of the research team during class time. The single blind procedure was applied to lower expectations and reactivity, and students were reminded that their participation was strictly voluntary. To reduce the social desirability bias, participants were also assured that their answers would be completely anonymous. None of the students refused to participate in the research project. The questionnaires were completed in a single session lasting between 20 and 30 min.

### Data Analysis

Missing values were calculated using the expectation maximization (EM) algorithm and the Markov chain Monte Carlo (MCMC), both offered by the LISREL 8.8 program. Outliers were also eliminated using the SAS program.

The following statistical programs were used: SPSS 25 for descriptive statistics and correlation coefficients; and EQS v.6.1 for testing the structural regression models.

The Structural Equations Models (SEM) method (specifically the complete structural regression model method) was used to test the hypothesized models. The analyses were carried out using the robust maximum likelihood (MLR) procedure, due to the deviation of the multinormal data (all Mardia’s normalized coefficient > 5, *p* < 0.01) (Bentler, 2005). Diverse indexes were used to test the models’ goodness of fit (Byrne, 2001): the Satorra-Bentler chi-square statistic (SBχ^2^) and the degrees of freedom, CFI (Comparative Fit Index), TLI (Tucker-Lewis Index) whose value must be higher than 0.90 (Barrett, 2007), and RMSEA (Root Mean Square Error of Approximation) of 0.06 or less with its confidence intervals (Hu and Bentler, 1999).

The use of relatively large samples implies problems in applying the hypothesis test as a criterion for choosing between alternative models (Bentler and Bonett, 1980). For this reason, apart from the Satorra-Bentler chi-square statistic (SBχ^2^) and the consecutive χ^2^/gl ratio that considers a score of 2.00–3.00 or less as good fit (Marsh and Hau, 1996), the ACI (Akaike information criterion) (Akaike, 1987) and CAIC (Consistent Akaike information criterion) (Bozdogan, 1987) are calculated in order to compare non-nested models and the lowest value indicates the greatest parsimony.

However, when comparing nested models χ^2^ significance tests are used. The principle to be applied is the following: (a) if the χ^2^ test result is significant, the model of choice would be the baseline or partial mediation model because its loss in *df* is justified by the significant improvement in model fit; and (b) if the χ^2^ test result is not significant, the full mediation model is to be accepted as it represents a more parsimonious model with comparable fit to the baseline or partial mediation model (Diamantopoulos and Siguaw, 2000).

## Results

### Descriptive Statistics and Correlations Between the Study Variables

Prior to analyzing the measurement model, a Pearson correlation analysis was conducted, along with an analysis of the means and standard deviations. The results are shown in Table 2.

**TABLE 2:** Bivariate zero-order correlations, means and standard deviations of the variables of the study.

Variables	1	2	3
1. Sociometric popularity	1	0.157**	0.048
2. Peer support		1	0.192**
3. General self-concept			1
Mean	3.52	3.98	3.90
SD	20.68	0.67	0.77

**p < 0.01.

### The Measurement Model

The measurement model includes three latent variables whose indicators are the items in the questionnaires administered. The measurement model analysis revealed an acceptable fit: SBχ^2^(60) = 119.97, *p* < 0.001; CFI = 0.964; TLI = 0.953; RMSEA = 0.039; RMSEA confidence interval 90% = 0.029–0.049). All factor loadings of the latent variable indicators were significant (*p* < 0.01), which implies that all latent factors are represented by their corresponding indicators.

### Analysis of the Hypothesized Models

Having analyzed the measurement model, the global fit was calculated for each of the different theoretical models tested (Figure 1) in order to verify the nature of the relationships between the variables in the study. The first regression model tested was *peer support* and *sociometric popularity* simultaneously on *general self-concept* (M_1_). Next to be tested was the full mediation model (M_2a_) between *peer support* and *general self-concept* through *sociometric popularity*, and the partial model (M_2b_), which posits a direct pathway from *peer support to general self-concept*. Finally, the full (M_3a_) and partial (M_3b_) mediation models between *sociometric popularity* and *general self-concept* through *peer support* were tested.

The initial hypothetical model (M_1_) posits that the variables *peer support* and *sociometric popularity* predict *general self-concept*. An initial analysis of the resulting parameters indicated that this model (M_1_) had a good fit: SBχ^2^(60) = 135.47, *p* < 0.001; CFI = 0.956; TLI = 0.943; RMSEA = 0.043; RMSEA confidence interval 90% = 0.033–0.053.

To verify the hypothesized models M_2a_ and M_2b_, the goodness of fit indexes for the full mediation model (direct pathway from *peer support* to *general self-concept* restricted to zero) were compared with those of the partial mediation model (direct pathway from *sociometric popularity* to *general self-concept*). Both models have a good fit: M_2a_ SBχ^2^(60) = 122.01, *p* < 0.001; CFI = 0.964; TLI = 0.953; RMSEA = 0.039; RMSEA confidence internal 90% = 0.029–0.049; and M_2b_ SBχ^2^(59) = 119.97, *p* < 0.001; CFI = 0.964; TLI = 0.953; RMSEA = 0.039; RMSEA confidence internal 90% = 0.029–0.049. The Chi-square test on the discrepancy between the two models [χ^2^(1) = 2.8302, *p* = 0.093] was found not to be statistically significant. In this case, the full mediation model is to be accepted as it represents a more parsimonious model with comparable fit to the baseline model.

The analysis of the goodness of fit indexes pertaining to the final two models tested revealed that both M_3a_ and M_3b_ had good levels: M_3a_ SBχ^2^(60) = 120.32, *p* < 0.001; CFI = 0.965; TLI = 0.954; RMSEA = 0.039; RMSEA confidence internal 90% = 0.028–0.048; and M_3b_ SBχ^2^(59) = 119.97, *p* < 0.001; CFI = 0.964; TLI = 0.953; RMSEA = 0.039; RMSEA confidence internal 90% = 0.029–0.049. The Chi-square test on the discrepancy between the two models [χ^2^(1) = 0.0151, *p* = 0.090] was not statistically significant. Between these two models, again the one chosen is the model of full mediation since it is more parsimonious.

The goodness of fit indexes found for all five models tested (Table 3) were good, but model M_3a_ had the best fit, obtaining the lower AIC and CAIC. The results suggest that M_3a_ is the most parsimonious and therefore the first-choice model.

**TABLE 3:** Adjustment indexes of full and partial mediation models.

Model	SBχ^2^_(gl)_	SBχ^2^/gl	CFI	TLI	RMSEA_(IC)_	AIC	CAIC
M_1_	135.47_(__60__)_	2.26	0.956	0.943	0.043_(__0_._033__–__0_._053__)_	15.471	−315.500
M_2a_ Full mediation	122.01_(__60__)_	2.03	0.964	0.953	0.039_(__0_._029__–__0_._049__)_	2.009	−328.963
M_2b_ Partial mediation	119.97_(__59__)_	2.03	0.964	0.953	0.039_(__0_._029__–__0_._049__)_	1.967	−323.488
M_3a_ Full mediation	120.32_(__60__)_	2.01	0.965	0.954	0.039_(__0_._028__–__0_._048__)_	0.324	−330.647
M_3b_ Partial mediation	119.97_(__59__)_	2.03	0.964	0.953	0.039_(__0_._029__–__0_._049__)_	1.967	−323.489

*SBχ^2^/gl < 3; CFI and TLI > 0.90 (acceptable fit) or > 0.95 (good fit); RMSEA < 0.05 (good fit); AIC and CAIC lower value.*

### Standardized Regression Coefficients

When the regression coefficients of the first-choice model (M_3a_) were analyzed individually (Table 4), all the direct pathways proposed were found to be significant at a significance level of *p* < 0.01.

**TABLE 4:** Standardized regression coefficients.

	Standardized beta
**Direct effects**	
Sociometric popularity → Peer support	0.870**
Peer support → General self-concept	0.157**
**Indirect effects**	
Sociometric popularity → General self-concept	0.136**

**p < 0.01; R^2^ (peer support) = 0.757; R^2^ (general self-concept) = 0.025.

**FIGURE 2:**
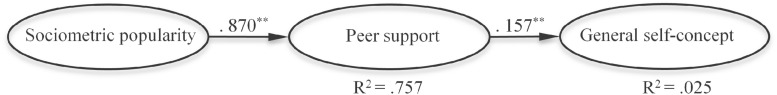
Final structural model.

Specifically, *sociometric popularity* was found to predict 76% of *peer support*, while the variables *sociometric popularity* and *peer support* determined 3% of *general self-concept*. For its part, the variable *sociometric popularity* indirectly determined *general self-concept*, while *peer support* was found to fully mediate between these two variables. The final structural model with its regression coefficients is shown in Figure 2.

## Discussion

The studies carried out on the role of peer support on the relationship between popularity and self-concept are non-existent, so there is a large gap in previous research that this study seeks to address. Consequently, this study tries to clarify the position that peer support occupies in the relationship of popularity with self-concept following the widely accepted Sociometer Theory (SMT).

The main aim of the education system, beyond transmitting knowledge and assessing basic academic competences, is to foster students’ social and emotional skills in order to ensure good psychosocial adjustment. This expanded role, coupled with the current view of schools as environments in which to promote healthy development, has generated a growing interest in the study of variables linked to better adjustment (Froh et al., 2011; Kristjánsson, 2012), including sociometric status (Inglés et al., 2017), the support provided in different contexts (parents, teachers and peers) and self-concept (Rodríguez et al., 2012; Rodríguez-Fernández et al., 2016a). Moreover, it has been demonstrated that during adolescence, adjustment in the school context is partly facilitated by the social and emotional support provided by peers (Rodríguez-Fernández et al., 2016a).

We can therefore conclude that a priority objective for any education system would be to include social relationships in the curriculum, to which end it is necessary to analyze and understand their diversity in terms of preferences, popularity, friendship, social networks and perceived social relations, etc. It is also vital to understand how the aforementioned variables are related to each other, which is why the present study analyzes the relationships which exist between sociometric popularity (measured objectively through peer acceptance rather than using self-reported questionnaires), perceived social support from peers and self-concept among adolescents.

The results obtained here confirm, consistently with the findings of previous studies, that perceived support from significant peers is a key variable in the level of self-concept attained during adolescence (Mruk, 2006; Kong et al., 2015; Magro et al., 2019), with those who perceive their friends to be more available and ready to help also having a better concept of themselves. Viewed from a negative perspective, this points to the importance of paying attention to the negative messages conveyed by classmates, since these seem to have a harmful effect on self-concept, particularly during adolescence, a period in which peer relations become more assiduous and messages received from friends have a greater impact than in later stages of life, making self-concept more sensitive to their influence (Ramos-Díaz et al., 2016).

One of the novel contributions made by this study is that it addresses the directionality of the relationship between popularity and peer support during adolescence. To date, research has either focused solely on aspects linked to friendship, such as number of friends, conflicts between friends or friendship understood as something reciprocal (Nangle et al., 2003; Rose et al., 2004), or has analyzed perceived popularity (Litwack et al., 2012), finding a positive relationship between these two variables. The results found here indicate that it is sociometric popularity that precedes perceived social support, rather than the other way round. Thus, being more or less popular, more or less rejected or more or less ignored affects students’ perceptions of the social support they receive from their peers. This is a finding that extends our previous knowledge, namely that the number of positive nominations received correlates positively with perceived support (Wentzel, 2003), since, in this case, receiving many positive nominations and few negative ones was found to increase the perception of social support.

The results also indicate that sociometric popularity has an indirect impact on adolescents’ general self-concept through perceived peer support. If the factors included in the model are compared, the direct effect of perceived peer support on self-concept is observed to be more intense than the indirect effect of sociometric popularity, thereby countering the idea that sociometric popularity has a direct effect on general self-concept, as believed until now (Litwack et al., 2012). Rather, sociometric popularity is revealed as having an indirect effect on self-concept through peer support. This is perhaps the study’s most important finding.

The study has a number of limitations that should be taken into account. Firstly, the results presented here were extracted using the structural equations method which provides information about the plausibility of hypothesized causal relationships (Ruiz et al., 2010), but which cannot, under any circumstances, confirm causality. Longitudinal studies are therefore required to determine the causal directionality of the relationships which exist between the variables studied, including the mediating effect of perceived peer support.

Furthermore, in order to enable the results to be generalized and to test the model obtained in a stricter manner, future studies should conduct a multi-sample analysis in accordance with sex and school year, focusing on both secondary school students and those studying at other educational levels. This would enable them to test whether the links observed are the same in men and women, and whether they are maintained throughout students’ academic careers. Moreover, the results obtained in this study may be further explored by distinguishing between different domains of self-concept (physical, social, personal and academic) or even by including other mediating contextual variables, such as support from teachers and family (Ramos-Díaz et al., 2016), and other psychological variables such as emotional intelligence or resilience, which have also been shown to be associated with adolescents’ positive development (Rodríguez-Fernández et al., 2016a).

## Conclusion

The results of this present study have important educational implications, one of the most significant being to highlight the key relevance of peer relations during adolescence, since the data indicate that the general self-concept of rejected students (i.e., those who received many negative and few positive nominations) is impacted by the mediating buffer effect of subjective perceptions of peer support. Since rejection is associated with school violence (Martínez-Ferrer et al., 2012), there is clearly a need for preventive interventions with the peer group. The results of this study suggest that interpersonal relationships with peers provide a good socialization context in which to learn and develop new social and emotional skills, which in turn will determine to what extent individuals perceive themselves in a positive or negative way, leading to greater or poorer adjustment to their environment (Rodríguez-Fernández et al., 2016a) and more or fewer recourses for preserving their psychological well-being (Rodríguez-Fernández et al., 2016b).

Thus, it is important to design and implement psychoeducational interventions aimed at fostering positive peer relations in the school context. This study has demonstrated that social support depends on acceptance or rejection by peers, and in turn has a key impact on self-concept, which points to the need for schools to provide adolescences with a good social support network in order to ensure their adequate psychological functioning. We should not forget either the importance of fostering positive self-perception and acceptance of oneself in secondary education, a period in which the level of positive self-perception tends to drop (Coelho and Romão, 2017; Van der Aar et al., 2018).

This study has shown that social support depends on the acceptance or rejection by peers and that this in turn has an effect, so it is concluded that it is necessary.

In summary, it is necessary to promote support networks that protect, provide affection and create a space where adolescents feel loved, valued, listened and understood. It is essential to listen to adolescents, respect them, believe in them, so that they can believe in themselves and trust in their own capacity to build a healthy life project for their psychosocial well-being (Orcasita and Uribe, 2010). It is a priority, in turn, to encourage positive interactions between peers by providing adolescents with resources and social skills appropriate to the improvement of such socialization processes that result in a more positive self-concept.

## Data Availability Statement

The datasets generated for this study are available on request to the main author.

## Ethics Statement

This study has received a favorable report from the University of the Basque Country’s Ethics Commission (CEISH/UPV-EHU, BOPV 32, EHAA, memory number M10/2015/076), stating that the study complied with the ethical values established for research with humans (informed consent, right to information, personal data protection, confidentiality guarantees, non-discrimination, non-remuneration and the right to withdraw at any time). Written informed consent to participate in this study was provided by the participants’ legal guardian/next of kin.

## Author Contributions

AF-Z, ER-D, and AR-F conceived the design and wrote the manuscript. AF-Z and ER-D conducted the data analysis. JN co-led the manuscript preparation. All authors have read and agreed to the published version of the manuscript. All authors contributed to the article and approved the submitted version.

## Conflict of Interest

The authors declare that the research was conducted in the absence of any commercial or financial relationships that could be construed as a potential conflict of interest.
